# Efficacy and safety of condylectomy with minimally invasive surgery in the treatment of interdigital corns of the lesser toes compared to conservative treatment

**DOI:** 10.1186/s13047-021-00460-0

**Published:** 2021-03-20

**Authors:** Luis M. Marti-Martinez, Rubén Lorca-Gutierrez, Salvador Pedro Sánchez-Pérez, Jonatan Garcia-Campos, Nadia Fernández Ehrling, Javier Ferrer-Torregrosa

**Affiliations:** 1 Behavioural and Health Sciences Department, Miguel Hernandez University, Ctra. Alicante-Valencia s/n, 03550 San Juan de Alicante, Spain; 2 Physiotherapy and Podiatry Department, Catholic university of Valencia San Vicente Mártir, Torrente, Spain

**Keywords:** Interdigital corn, Minimal invasive surgery, Foot, Heloma

## Abstract

**Background:**

Minimally invasive surgery (MIS) procedures cause less trauma to the patient and might improve recovery. This study aimed to determine the efficacy and safety of condylectomy with MIS to treat interdigital corns of the lesser toes.

**Methods:**

This prospective cohort study was conducted in seven podiatry centers. Patients with interdigital corns of the lesser toes, progressing for more than a year, with one or more recurrences in the last year following conservative treatments were eligible. The recruited patients were classified according to their treatment: conservative or surgical (condylectomy with MIS) and were compared. Patient satisfaction, pain, the clinical and functional status of the foot and the appearance of sequelae were assessed at 3 and 6 months after treatment.

**Results:**

At 6 months, patients in the surgical treatment group showed no pain on pressure, which significantly differed from the conservative treatment group (*p* <  0.001). They also improved clinical and functional status of the foot, reaching values comparable to those of the standard population. No paresthesia, joint stiffness or instability, toe malalignment, or corn transfer to a contiguous site resulted from the surgical treatment.

**Conclusions:**

Condylectomy with MIS is effective and safe to treat interdigital corns of the lesser toes.

**Supplementary Information:**

The online version contains supplementary material available at 10.1186/s13047-021-00460-0.

## Background

An interdigital corn of the lesser toes is a circumscribed hyperkeratotic lesion resulting from epidermal hyperplasia, specifically that involving the stratum corneum [1] (Additional figures 1 and 2). According to du P Gillett [2], 15% of individuals presenting with some type of foot disease have interdigital corns of the lesser toes. These types of corns are most frequently observed in elderly patients; a higher prevalence in observed women than in men, possibly due to the characteristics of their footwear [3, 4]. An interdigital corn is painful under pressure, and although the intensity of pain is variable, the interdigital corn will invariably make it difficult or impossible to put on shoes and walk normally [5]. This condition is often associated with inflammation, and ulceration or infection may occur in severe cases [6].

The treatment of interdigital corns in the foot aims to relieve symptoms in addition to addressing the mechanical etiology [6], and should eliminate the mechanical pressure on the skin. This treatment can be conservative; however, if such treatment fails and recurrence and pain are observed, surgical treatment is administered. Conservative treatments can be noninvasive (mechanical debridement with a scalpel, application of keratolytic, interdigital separators, insoles, adapted footwear) [5, 6] or invasive (injection of fillers such as collagen [7]). Different surgical alternatives are available to treat interdigital corns: condylectomy, arthroplasty, syndactyly [5, 8]. If a forefoot osteoarticular deformity causes digital deformities, such as claw toe, the deformity should be corrected, previously, using the same surgery option chosen for the treatment. In the case of treatment with condylectomy, the deformity should be corrected with minimally invasive surgery (MIS) by incomplete phalanx osteotomy without tenotomy [9].

Currently, the trend worldwide in any surgical treatment is to develop and investigate minimally invasive procedures which cause less trauma to the patient and consequently improve recovery [10]. The surgical technique of condylectomy with MIS for the treatment of interdigital corns of the lesser toes has been performed by podiatric and orthopedic surgeons for several decades [11]. However, its efficacy and safety must be demonstrated to recommend its use.

## Methods

The objective of the present study was to determine the efficacy and safety of condylectomy with MIS using a bur in the treatment of interdigital corns of the lesser toes.

This observational, prospective, and multicenter cohort study was conducted in seven private podiatry centers in Spain (two centers in Catalonia, one center in La Rioja, one center in the Basque Country, one center in Madrid, one center in Castilla-La Mancha, and one center in Murcia) from February 15 to December 15, 2016, in accordance with the Declaration of Helsinki.

Patients who voluntarily visited the participating centers from February 15 to April 15, 2016, and who met the inclusion criteria were consecutively invited to participate in this study. The following inclusion criteria were used: patients older than 18 years with interdigital corns of the lesser toes, progressing for more than a year, with one or more recurrences in the last year following conservative treatments, and those who were referred for conservative or surgical treatment for interdigital corns of the lesser toes. Patients who had previously been subjected to surgery of the same interdigital corn, those subjected to other surgical procedures on the same foot, those with mental disorders, and those who did not sign the informed consent form were excluded from the study.

A podiatric surgeon with more than 10 years of experience from each center was the researcher responsible for including study patients, collecting data at all follow-up visits, and performing treatments. The patients were invited to participate in the study after the interdigital corn treatment had been selected based on podiatrist criteria and patient preference. The recruited patients were classified according to their treatment (conservative or surgical treatment group). All patients returned for follow-up visits at 3 and 6 months after their treatment.

All the podiatry centers that participated in this study regularly performed condylectomy with MIS as surgical treatment for interdigital corns of the foot, and corn exfoliation with a scalpel, interdigital separators, or application of chemical keratolytic as conservative treatments.

### Surgical treatment

All podiatric surgeons performed condylectomy with MIS as described in a previous study [11]. If there was an osteoarticular deformity associated with interdigital corn, such as claw toe or hallux valgus, the deformity was corrected at the same moment of condylectomy using MIS by incomplete phalanx osteotomy without tenotomy. A local anesthesia technique was used for the affected toe and the surgery was performed without exsanguination of the toe. A longitudinal incision to the distal pulp of the toe or an incision to the center of the plantar aspect of the head of the proximal phalanx was performed according to the affectation (Additional figure 3). A Beaver 64 scalpel blade was used to make a stab incision and deepened until contact with the bone (Additional figure 4). A blunt elevator was used to separate adhesions and periosteal elevation (Additional figures 5 and 6). A Shannon-Isham burr of appropriate size was used to perform an osteotripsy (Additional figure 7). The bone paste was extracted with a rasp. Finally, the closure of the incision was performed with a suture thread (Additional figures 6 and 7). The fluoroscope was used as an intraoperative control element of the surgical technique due to its low emission of radiation compared with conventional X-rays, following the recommended precautions for radiology. The surgery method used was repeated at all study centers.

### Conservative treatment

Each conservative treatment was applied based on the clinical criteria and treatment preferences of the podiatrist responsible for the center. However, in each conservative treatment, the same instrumentation was used at all centers. For mechanical debridement, a scalpel consisting of a number 15 blade inserted into a number 3 handle was used; as an interdigital separator, a custom-made silicone elastomer interdigital orthosis was used; and as a chemical keratolytic, the product Quocin® was used according to the manufacturer’s instructions.

### Study variables

At the first visit, before treatment initiation, data on the following variables were collected: age, sex, description of the anatomical location of the interdigital corn (foot, toe, and phalanx), presence of an interdigital corn in more than one toe (no / yes, on 2nd toe/ yes, on 3rd toe/ yes, on 4th toe/ yes, on 5th toe), presence of paresthesia (yes/no), presence of condyle hypertrophy or exostosis on radiographic examination (yes/no), presence of pain on pressure in the area of the interdigital corn (yes/no), treatment (surgical/conservative), and when applying conservative treatment, the type of conservative treatment (exfoliation /separation /chemical treatment).

To determine the treatment efficacy, the following parameters were evaluated:

(i) degree of patient satisfaction with the treatment was assessed through a structured interview and using the categories outlined in Additional Table 1 (grouped into ‘Poor-fair-good’, ‘Very good or Excellent’ for better statistical treatment of the data);
(ii) interdigital corn recurrence (yes/no), and if recurrent, whether recurrence was ‘similar’ or ‘smaller’;
(iii) pain on pressure in the area of the interdigital corn (yes/no);
(iv) degree of pain was measured using a visual analog scale (VAS) ranging from 0 to 10 (0 being the absence of pain and 10 maximum pain), and using the ‘pain’ dimension of the American Orthopedic Foot and Ankle Society (AOFAS) scale [12], ranging from 0 to 40 (0 being maximum pain and 40 absence of pain);
(v) foot function was evaluated using the ‘function’ dimension of the AOFAS scale, ranging from 0 to 45 (0 being maximum limitation and 45 being maximum function). The joint motion was empirically evaluated by the same podiatrist before and after the treatment;
(vi) clinical and functional status of the foot was assessed using the total score on the AOFAS scale, ranging from 0 to 100 (0 being the worst result and 100 the best).

All these variables were measured before treatment, and at 3 and 6 months after treatment, except for satisfaction and recurrence which were only measured during posttreatment visits.

To determine the safety of the treatment, the appearance of sequelae, and the type and incidence of the appearance of sequelae were assessed at 3 and 6 months after administering the treatment. The following variables were evaluated:

(i) presence of paresthesia (yes/no),
(ii) interdigital corn transfer (yes/no). This study variable was included to analyze the possibility that the removed pressure from one condyle could be transferred to the other condyle when the condylectomy was excessively aggressive;
(iii) alignment of the toe with the interdigital corn, based on the ‘alignment’ dimension of the AOFAS scale which ranged from 0 to 15 (0 being poor alignment and 15 good);
(iv) metatarsophalangeal (MTP) joint motion of the toe with the interdigital corn, based on its dimension of the AOFAS scale which ranged from 0 to 15, (0 being the worst and 15 the best MTP joint motion);
(v) interphalangeal joint (IPJ) motion of the toe with the interdigital corn, based on its dimension of the AOFAS scale which ranged from 0 to 5 (0 being the worst and 5 the best IPJ motion);
(vi) degree of MTP joint-IPJ stability of the toe with the interdigital corn, based on the AOFAS scale which ranged from 0 to 5 (0 being unstable or dislocatable and 5 being stable).

Posttreatment interdigital corn transfer was the only variable assessed at 3 and 6 months after treatment. The other variables were also measured before the treatment because a worsened score of the variable at 3 and/or 6 months would indicate that the surgical treatment had caused this unwanted effect. Other complications like prolonged swelling (duration of more than 1 month), wound problems or infections were also registered.

### Statistical analysis

The minimum sample size was calculated based on clinical experience, considering 40% no recurrence for the conservative treatment group and 80% for the surgical treatment group. Accepting an alpha risk of 0.05 and a beta risk of 0.2 in a bilateral contrast, 24 patients who were applied conservative treatment and 24 patients who were applied the surgical treatment were required to detect the difference between the two proportions as statistically significant. A loss to follow-up rate of 5% was expected and the arcosene approximation was used.

Univariate analysis was performed for all variables; the number and percentage for qualitative variables and the minimum, maximum, mean, and 95% confidence intervals (CI) for quantitative variables were calculated. To compare qualitative variables, double-entry tables were constructed, applying the Chi-Squared test. For quantitative variables, the mean scores of the variables between two treatments were compared at each of the three time points, using the Student’s t-test. Subsequently, the non-parametric Friedman test was used to compare the three mean scores for each treatment assessed at the three time points. Differences whose probability of being due to chance was lower than 5% (*p* <  0.05) were accepted as significant. The statistical program SPSS v.18 for Windows was used for analysis (SPSS Inc., Chicago, Illinois (USA)).

## Results

A total of 59 patients were included in and completed the study, with no follow-up losses. Table 1 outlines the baseline characteristics of the sample; 89.8% (*n* = 53) of the population were women. The mean age of the study patients was 62.2 years, ranging from 30 to 84 years. All study patients presented with pain on mechanical pressure in the area of the interdigital corn before treatment.

**Table 1:** Baseline characteristics of the sample

	n	%
**Age**		
< 55 years	16	27.1
55–74 years	31	52.5
> 74 years	12	20.3
**Sex**		
Male	6	10.2
Female	53	89.8
**Treatment**		
Conservative (exfoliation)	20	33.9
Conservative (chemical)	0	0.0
Conservative (interdigital separator)	8	13.6
Surgical	31	52.5
**Foot with the interdigital corn**		
Right	39	66.1
Left	16	27.1
Bilateral	4	6.8
**Toe with the interdigital corn**		
2nd toe	12	20.3
3rd toe	4	6.8
4th toe	8	13.6
5th toe	35	59.3
**Phalanx with the interdigital corn**		
Distal	27	45.8
Proximal	23	39.0
Medial	1	1.7
More than one phalanx	8	13.6
**Pretreatment condyle hypertrophy or exostosis**		
Yes	54	91.5
No	5	8.5
**Interdigital corn in more than one toe**		
No	47	79.7
Yes, 2nd toe	0	0.0
Yes, 3rd toe	2	3.4
Yes, 4th toe	8	13.6
Yes, 5th toe	2	3.4

### Treatment efficacy

At 6 months posttreatment, the degree of satisfaction was “very good or excellent” in 100% (*n* = 31) of the patients who underwent surgical treatment, in contrast to that in 28.6% (*n* = 8) of patients who underwent conservative treatment (Table 2). Table 2 shows recurrence and pain on pressure at 3 and 6 months after treatment for both groups.

**Table 2:** Patient satisfaction with treatment, interdigital corn recurrence, and pressure pain by treatment at each time point

	Conservative treatment		Surgical treatment		
	n	%	n	%	***p***-value
Satisfaction at 3 months					
Poor-Fair-Good	18	64.3	3	9.7	< 0.001*
Very good	7	25.0	13	41.9	
Excellent	3	10.7	15	48.4	
Satisfaction at 6 months					
Poor-Fair-Good	20	71.4	0	0.0	< 0.001*
Very good	4	14.3	7	22.6	
Excellent	4	14.3	24	77.4	
Recurrence at 3 months					
No	6	21.4	30	96.8	< 0.001*
Yes	22	78.6	1	3.2	
Recurrence at 6 months					
No	5	17.9	31	100.0	< 0.001*
Yes	23	82.1	0	0.0	
Pain on pressure at pretreatment					
No	0	0.0	0	0.0	–
Yes	28	100.0	31	100.0	
Pain on pressure at 3 months					
No	5	17.9	27	87.1	< 0.001*
Yes	23	82.1	4	12.9	
Pain on pressure at 6 months					
No	6	21.4	31	100.0	< 0.001*
Yes	22	78.6	0	0.0	

* <  0.05

**Fig. 1:**
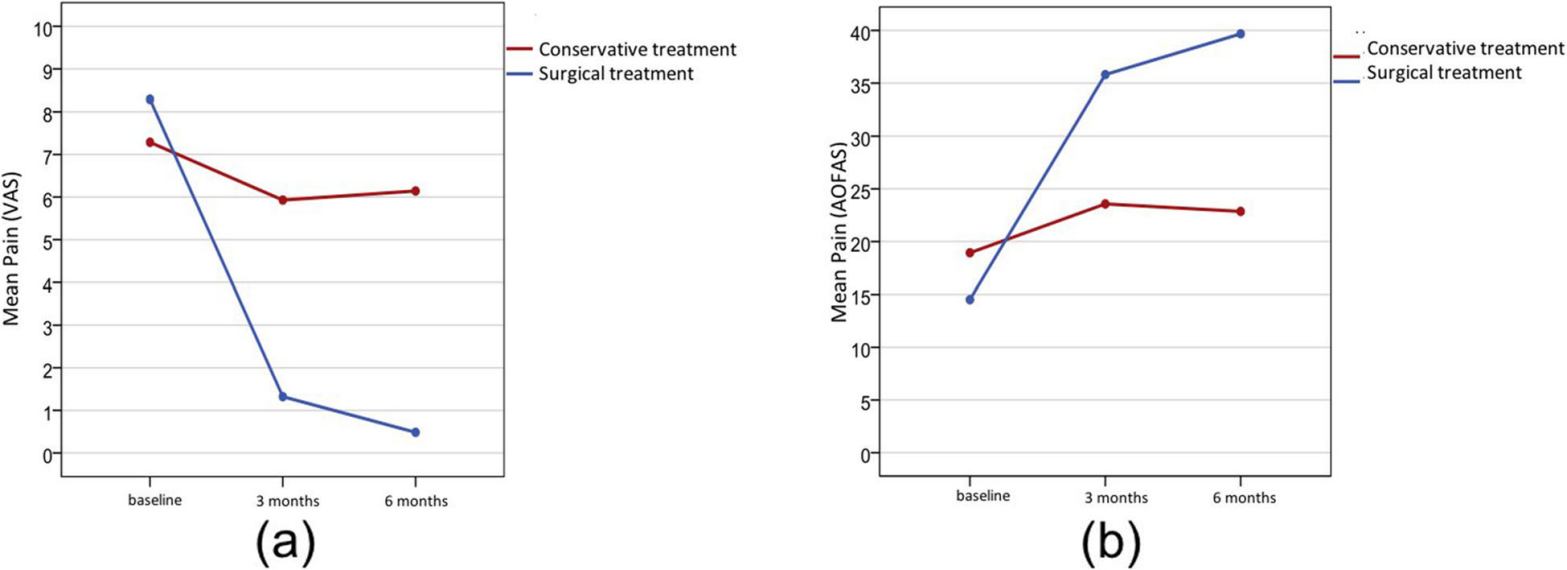
**a** Evolution of pain (VAS) over time; **b** Evolution of pain (AOFS) over time

Table 3 shows the comparison between treatment groups regarding pain according to the VAS and the AOFAS scale, before and at 3 and 6 months after treatment. Over time, in both treatment groups, the pain measured using the VAS (*p* = 0.016 for conservative treatments and *p* <  0.001 for surgical treatment) and the AOFAS scale (*p* = 0.001 for conservative treatments and *p* < 0.001 for surgical treatment) decreased significantly. In the group of patients who received surgical treatment, pain decreased sharply at 3 months. Pain parameters decreased even further at 6 months, reaching scores of 0.5 with almost no pain and of 40 with the total absence of pain, according to the VAS and the AOFAS scale, respectively (Fig. 1).

**Table 3:** Pain measured using a visual analog scale (VAS) and pain measured using the American Orthopedic Foot and Ankle Society (AOFAS) scale by treatment, at each time point

	n	Min.	Max.	Mean	95% CI	*p*-value
Pain (VAS) pretreatment						
Conservative treatment	28	5.0	9.0	7.3	(6.7–7.8)	< 0.003*
Surgical treatment	31	6.0	10.0	8.3	(7.9–8.6)	
Pain (VAS) at 3 months						
Conservative treatment	28	0.0	10.0	5.9	(4.8–7.0)	< 0.001*
Surgical treatment	31	0.0	6.0	1.3	(0.6–2.0)	
Pain (VAS) at 6 months						
Conservative treatment	28	0.0	10.0	6.1	(5.0–7.3)	< 0.001*
Surgical treatment	31	0.0	4.0	0.5	(0.1–0.9)	
Pain (AOFAS) at pretreatment						
Conservative treatment	28	0.0	30.0	18.9	(15.1–22.8)	0.088
Surgical treatment	31	0.0	30.0	14.5	(11.0–18.0)	
Dolor (AOFAS) at 3 months						
Conservative treatment	28	0.0	40.0	23.6	(18.6–28.5)	< 0.001*
Surgical treatment	31	30.0	40.0	35.8	(34.0–37.6)	
Dolor (AOFAS) at 6 months						
Conservative treatment	28	0.0	40.0	22.9	(17.8–27.9)	< 0.001*
Surgical treatment	31	30.0	40.0	39.7	(39.0–40.3)	

*CI* confidence interval, *VAS* visual analog scale, *AOFAS* American Orthopedic Foot and Ankle Society.

* < 0.05

**Fig. 2:**
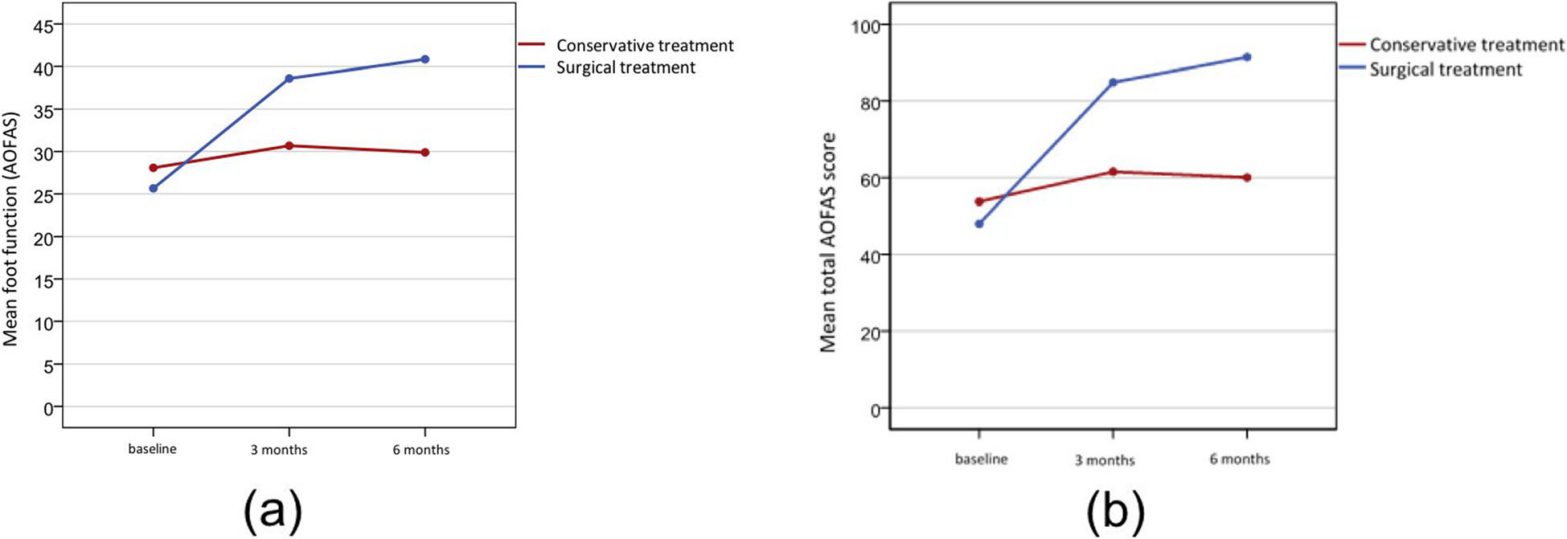
**a** Evolution of foot function (AOFAS score) over time.; **b** Evolution of total AOFAS score over time

Table 4 outlines the scores assessed in the dimension ‘function’ of the AOFAS scale and the total AOFAS score assessed in the treatment group before and after treatment. At baseline, no significant differences in foot function were observed between groups. However, in the surgical treatment group, foot function was significantly better than that in the conservative treatment group at three (*p* = 0.002) and six (*p* < 0.001) months after treatment. Figure 2a shows that both treatments significantly increased foot function over time. However, foot function remained unchanged at 3 months posttreatment in the group of patients who received conservative treatment, whereas it improved even further after 6 months in the surgical treatment group.

**Table 4:** Function dimension assessed by the American Orthopedic Foot and Ankle Society (AOFAS) scale and total AOFAS score by treatment, at each time point

	n	Min.	Max.	Mean	95% CI	***p***-value
Function at pretreatment						
Conservative treatment	28	4.0	40.0	28.1	(24.3–31.9)	0.291
Surgical treatment	31	0.0	37.0	25.6	(22.8–28.5)	
Function at 3 months						
Conservative treatment	28	0.0	45.0	30.7	(26.4–34.9)	0.002*
Surgical treatment	31	17.0	45.0	38.6	(36.3–40.9)	
Function at 6 months						
Conservative treatment	28	4.0	45.0	29.9	(25.7–34.1)	< 0.001*
Surgical treatment	31	17.0	45.0	40.8	(38.7–42.9)	
Total AOFAS score at pretreatment						
Conservative treatment	28	4.0	85.0	53.8	(45.6–62.0)	0.233
Surgical treatment	31	0.0	70.0	48.0	(42.4–53.6)	
Total AOFAS score at 3 months						
Conservative treatment	28	0.0	93.0	61.5	(51.9–71.2)	< 0.001*
Surgical treatment	31	47.0	100.0	84.9	(81.0–88.8)	
Total AOFAS score at 6 months						
Conservative treatment	28	4.0	93.0	60.0	(50.2–59.8)	< 0.001*
Surgical treatment	31	47.0	100.0	91.4	(87.9–94.9)	

*CI* confidence interval, *AOFAS* American Orthopedic Foot and Ankle Society.

* < 0.05

Regarding the clinical and functional status of the foot (AOFAS scale), no significant differences were found between groups at pretreatment (Table 4). At 3 and 6 months after treatment, the mean total score of the surgically treated patients was significantly higher (*p* < 0.001) than that of the patients who received conservative treatment (Table 4). Figure 2b shows that at 3 months posttreatment, significant improvements were observed in both the surgical (*p* < 0.001) and conservative (*p* < 0.001) treatment groups; at 6 months, in the surgical treatment group, in addition to the improved clinical and functional status of the foot, the patient scores reached values comparable to those of the standard population.

### Treatment safety

No patient in the conservative treatment group had paresthesia either before or after 3 months of treatment. However, 3.6% (*n* = 1) of the patients of this group had paresthesia at 6 months posttreatment. The percentage of patients from the surgical treatment group who had paresthesia decreased from 12.9% (*n* = 4) at pretreatment to 3.2% (*n* = 1) at 3 months posttreatment, and this reduction was maintained at 6 months posttreatment. Therefore, paresthesia was not a sequela of the surgical treatment.

In this study, 100% of the patients who received surgical treatment showed no posttreatment interdigital corn transfer; in contrast, in the group of patients who received conservative treatment, posttreatment interdigital corn transfer was observed in 3.6% (*n* = 1) and 7.1% (*n* = 2) of the cases at 3 and 6 months posttreatment, respectively. Posttreatment interdigital corn transfer, therefore, was not a sequela of the surgical treatment.

**Fig. 3:**
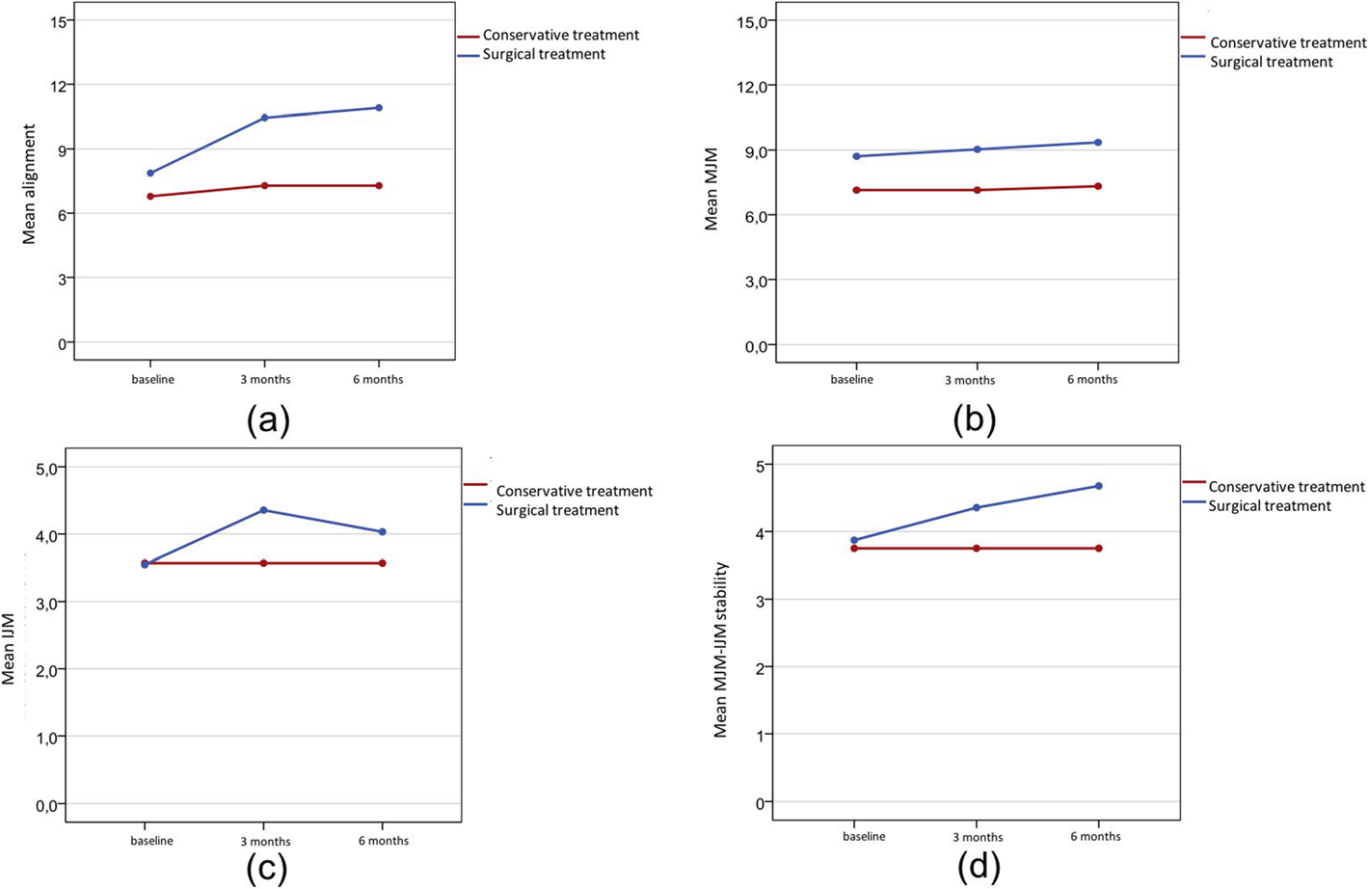
**a** Evolution of the alignment of the toe with the interdigital corn (AOFAS score) over time; **b** Evolution of the metatarsophalangeal joint motion (AOFAS score) over time; **c** Evolution of the interphalangeal joint motion (AOFAS score) over time; **d** Evolution of the stability score (AOFAS) over time

Regarding the alignment of the toe with the interdigital corn, at pretreatment, no significant differences were found between groups. Table 5 outlines the ‘alignment’ scores of both groups before and at 3 and 6 months after treatment. At 6 months, the scores of surgically treated patients were significantly higher (*p* = 0.002) than those of patients who received conservative treatment. In the conservative treatment group, the mean score for the alignment of the toe with the interdigital corn showed no significant changes (*p* = 0.135) over time (Fig. 3a). In turn, in the surgical treatment group, a significant improvement was observed at 3 months posttreatment (*p* < 0.001), which was maintained at 6 months posttreatment as well. Therefore, surgical treatment did not worsen the alignment of the toe with the interdigital corn.

**Table 5:** Alignment, metatarsophalangeal (MTP) joint motion, interphalangeal joint (IPJ) motion, and metatarsophalangeal joint- interphalangeal joint (MTPJ-IPJ) stability by treatment, at each time point

	n	Min.	Max.	Mean	95% CI	***p***-value
Alignment at pretreatment						
Conservative treatment	28	0.0	15.0	6.8	(5.2–8.4)	0.297
Surgical treatment	31	0.0	15.0	7.9	(6.4–9.3)	
Alignment at 3 months						
Conservative treatment	28	0.0	15.0	7.3	(5.5–9.1)	0.006*
Surgical treatment	31	0.0	15.0	10.5	(9.0–11.9)	
Alignment at 6 months						
Conservative treatment	28	0.0	15.0	7.3	(5.5–9.1)	0.002*
Surgical treatment	31	0.0	15.0	10.9	(9.4–12.4)	
MTP joint motion at pretreatment						
Conservative treatment	28	0.0	10.0	7.1	(5.7–8.6)	0.078
Surgical treatment	31	0.0	10.0	8.7	(7.7–9.8)	
MTP joint motion at 3 months						
Conservative treatment	28	0.0	10.0	7.1	(5.7–8.6)	0.026*
Surgical treatment	31	0.0	10.0	9.0	(8.2–9.9)	
MTP joint motion at 6 months						
Conservative treatment	28	0.0	10.0	7.3	(5.9–8.8)	0.015*
Surgical treatment	31	0.0	10.0	9.4	(8.6–10.1)	
IPJ motion at pretreatment						
Conservative treatment	28	0.0	5.0	3.6	(2.7–4.5)	0.970
Surgical treatment	31	0.0	5.0	3.5	(2.7–4.4)	
IPJ motion at 3 months						
Conservative treatment	28	0.0	5.0	3.6	(2.7–4.5)	0.147
Surgical treatment	31	0.0	5.0	4.4	(3.7–5.0)	
IPJ motion at 6 months						
Conservative treatment	28	0.0	5.0	3.6	(2.7–4.5)	0.415
Surgical treatment	31	0.0	5.0	4.0	(3.3–4.8)	
MTPJ-IPJ stability at pretreatment						
Conservative treatment	28	0.0	5.0	3.7	(2.9–4.6)	0.831
Surgical treatment	31	0.0	5.0	3.9	(3.1–4.7)	
MTPJ-IPJ stability at 3 months						
Conservative treatment	28	0.0	5.0	3.7	(2.9–4.6)	0.241
Surgical treatment	31	0.0	5.0	4.4	(3.7–5.0)	
MTPJ-IPJ stability at 6 months						
Conservative treatment	28	0.0	5.0	3.7	(2.9–4.6)	0.057
Surgical treatment	31	0.0	5.0	4.7	(4.2–5.0)	

*CI* confidence interval, *MTPJ* metatarsophalangeal joint, *IPJ* interphalangeal joint

* < 0.05

Regarding the level of MTP joint motion of the toe with the interdigital corn, there was no significant difference between groups before treatment (*p* = 0.078), as can be seen from Table 5. At 3 and 6 months posttreatment, MTP joint motion was significantly better in the surgical treatment group than in the conservative treatment group (Table 5). In both the conservative and surgical treatment groups, no significant improvements were observed (*p* = 0.368 and *p* = 0.082, respectively) in the MTP joint motion of the toe with the interdigital corn after the treatment (Fig. 3b). Therefore, the surgical technique did not affect the MTP joint motion of the toe with the interdigital corn.

Regarding the IPJ motion of the toe with the interdigital corn, significant differences were found between groups at any time point (Table 5). In the group of patients who received conservative treatment, the IPJ motion scores did not change after treatment. However, in the group of surgically treated patients, the mean IPJ motion score increased significantly (*p* = 0.022) (Fig. 3c). Therefore, the surgical technique did not affect the IPJ motion of the toe with the interdigital corn. Similarly, the degree of MTP joint-IPJ stability of the toe with the interdigital corn showed no significant differences between groups, either before or after treatment (Table 5). At 3 and 6 months after treatment, the stability score increased significantly only in the surgical treatment group (*p* = 0.042) (Fig. 3d). Therefore, the surgical technique did not impair the MTP joint-IPJ stability of the toe with the interdigital corn. Study patients had no other complications after treatment.

## Discussion

The results from the present study indicate the efficacy of the surgical technique and show that surgery eliminates interdigital corns and pain in this area in almost 100% of the cases. In this study, surgery improved the clinical and functional parameter values of the foot to levels comparable to normal values. In addition, the results also show that no paresthesia, joint stiffness or instability, toe malalignment, or interdigital corn transfer to a contiguous site resulted from the surgical treatment. Thus, this study indicates that performing condylectomy with MIS as per the protocol described here is safe for treating interdigital corns.

A review of the literature showed that there is a lack of studies assessing the efficacy of MIS in the treatment of interdigital corns. Coughlin et al. [13] studied the efficacy of condylectomy-based open surgery for interdigital corns in a series of patients. However, they only studied the interdigital corns that appeared in the fourth interdigital space. The study did not include a comparator group with a different treatment, and the authors indicated that the patients had previously undergone conservative treatment without success. In addition, due to its retrospective nature, some of the variables of interest in that study were only measured after the treatment; in some cases, measurements were obtained up to 7 years after applying the treatment.

As in Coughlin et al. [13], in the present study, the surgical treatment achieved an excellent or very good degree of satisfaction in all cases at 6 months posttreatment, which was higher than the degree of satisfaction achieved with the conservative treatment. In contrast to the conservative treatment group, the surgical treatment group reported no interdigital corn recurrences in any of the cases, both in the present study and in that by Coughlin et al. [13]. Regarding pain, the results from the present study show that surgical treatment more effectively reduces pain than does conservative treatment. Although the posttreatment perceived pain score was higher in the present study than in the study by Coughlin et al. [13], this parameter is not comparable due to the retrospective nature of the study by Coughlin et al. Although we cannot compare the results, condylectomy with MIS as a treatment for interdigital corns of the lesser toes improved foot function, and restored the functional and clinical status of the foot to a fully normal state. Therefore, based on our findings, condylectomy with MIS is an effective technique in the treatment of such interdigital corn cases. Thus, when the primary cause of mechanical stress on the skin is bone, conservative treatments will only be palliative, and not curative treatments.

Regarding the safety of their surgical treatment, Coughlin et al. [13] identified postsurgical paresthesia in 3.7% of the cases, which indicates that the treatment injured the nerves that are lodged in the affected toes. Furthermore, toe malalignment was observed in 3.2% of the cases because one of the surgical techniques they used was IPJ arthroplasty, which can affect toe position and IPJ motion. In fact, in 16% of the cases, they identified increased rigidity after applying the treatment. In the present study, the percentage of patients with paresthesia improved after applying the surgical treatment, most likely because pretreatment paresthesia was derived from inflammatory responses triggered by condyle hypertrophy and the interdigital corn. Accordingly, when resolving the interdigital corn, the paresthesia improved. In turn, the MIS technique is based on condylectomy; hence, the position of the IPJs is not changed and therefore the alignment of the toe did not change either. It is thus possible that the elimination of this pain generated a non-antalgic position, thereby improving the alignment of the affected toe. For reasons that are yet unclear, a small, albeit significant improvement in IPJ motion and in MTP-IP joint stability was observed in the group of patients who received treatment with MIS. Since no treatment was applied to the MTP, no change in MTP motion was expected, as observed in both groups of the present study. Therefore, MIS for interdigital corns is safer when compared with open surgery, because of the following reasons: it reduces the risk of triggering neurological injury to the toe in question, avoids posttreatment interdigital corn transfer, and is not associated with sequelae such as malalignment of the affected toe, and altered joint motion or stability.

### Strengths and limitations of the study

Due to the multicenter nature of the study, the generalizability of the results is high. The participation of experienced podiatric surgeons ensured the reproducibility of the surgical technique. Regarding the main limitations of the study, the final decision on the treatment was taken by the patient; as a result, a higher percentage of patients with a more painful and exacerbated interdigital corn may have opted for treatment with MIS surgery, seeking a more definitive solution to their ailment. This may have resulted in a less homogeneous distribution in the groups. In addition, podiatric surgeons, who knew the treatment of the patient, assessed the study outcomes; therefore, podiatrists might have influenced some study variables. Another limitation that needs to be highlighted when analyzing the conclusions from this study is the cohort follow-up time. It is unclear whether the results from the analysis of the study variables can be maintained in the long term. And, lastly, a measurement bias could exist due to the joint motion was empirically evaluated without using a more accurate measurement. The same podiatrist measured this parameter before and after the patient treatment to correct this bias.

## Conclusions

Condylectomy with MIS using a bur of the involved bone surface is an effective and safe treatment for recurrent interdigital corns of the lesser toes, and should be considered as an alternative approach to treat recurrent interdigital corns. Although the results of the present study show differences between conservative and surgical treatments, we should be cautious when interpreting these findings because the study objective was to determine the efficacy and safety of condylectomy with MIS and not the comparison of treatments. Further research, specifically experimental studies, is needed to compare the efficacy and safety between conservative and surgical treatments.

## Supplementary Information

**Additional file 1: Figure S1.** Heloma on distal phalanx on fifth toe.

**Additional file 2: Figure S2.** Heloma on head proximal phalanx on fourth toe.

**Additional file 3: Figure S3.** Condylectomy incision.

**Additional file 4: Figure S4.** Condilectomy incision with Beaver 64 scalpel blade.

**Additional file 5: Figure S5.** Blunt elevator to separate adhesions.

**Additional file 6: Figure S6.** Fluoroscope image of separation of adhesions.

**Additional file 7: Figure S7.** Osteotripsy with Shannon-Isham burr.

**Additional file 8: Figure S8.** Fluoroscope image before condylectomy.

**Additional file 9: Figure S9.** Fluoroscope image after condylectomy.

**Additional file 10: Table S1.** Categories used to evaluate the degree of patient satisfaction with the treatment, based on a structured interview.

## Data Availability

The datasets used and/or analyzed during the current study are available from the corresponding author on reasonable request.

## Abbreviations

MIS: Minimally invasive surgery
VAS: Visual analog scale
AOFAS: American Orthopedic Foot and Ankle Society
MTP: Metatarsophalangeal
IPJ: Interphalangeal joint
CI: Confidence interval
