# Supplementary figures and images for: Efficacy and safety of condylectomy with minimally invasive surgery in the treatment of interdigital corns of the lesser toes compared to conservative treatment
Source: J Foot Ankle Res. 2021 Mar 20;14:20. doi: 10.1186/s13047-021-00460-0 (PMC7981973; doi:10.1186/s13047-021-00460-0)

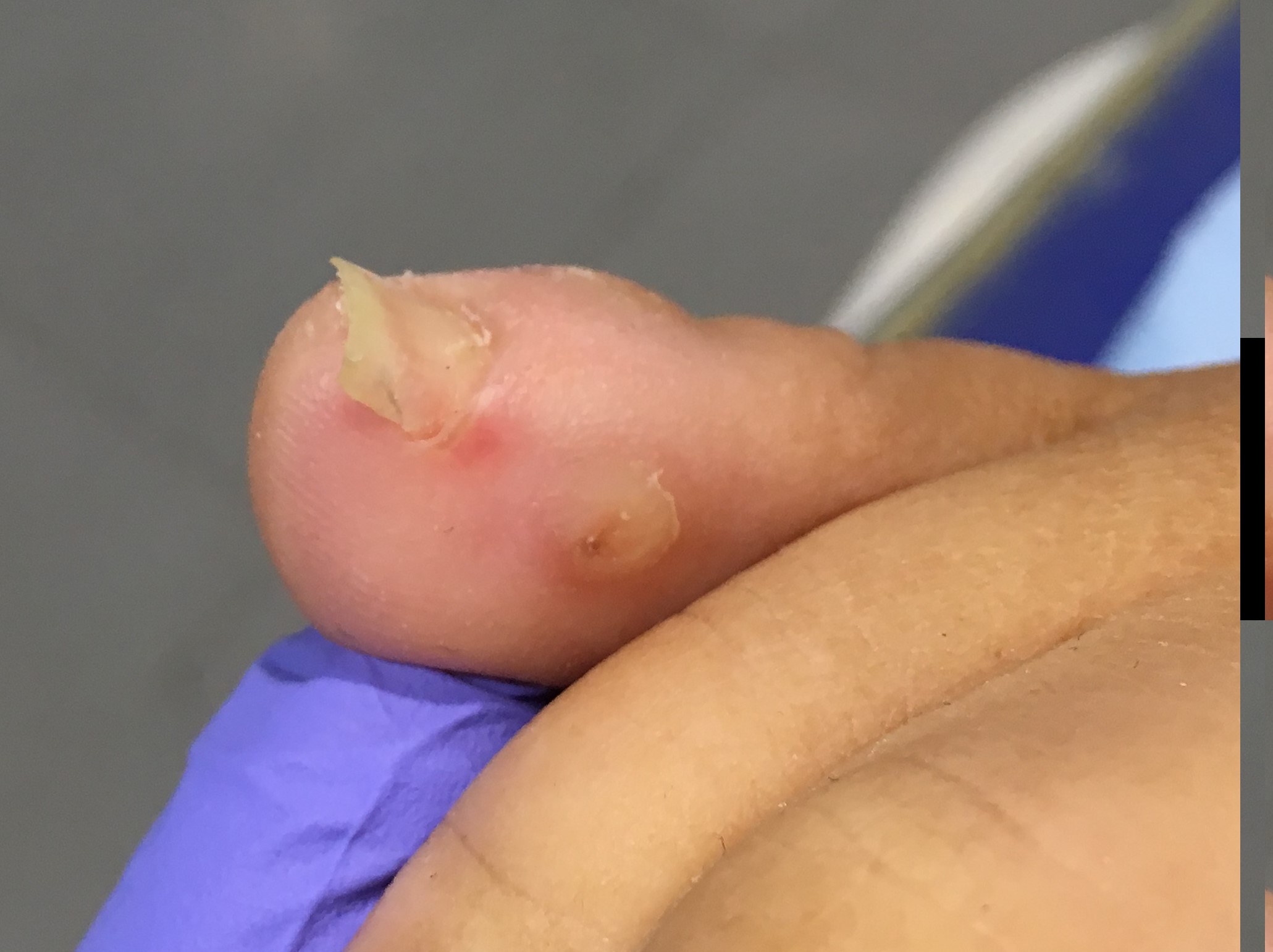

Supplement: Supplementary file 1 — Additional file 1: Figure S1. Heloma on distal phalanx on fifth toe. [file 13047_2021_460_MOESM1_ESM.jpg]

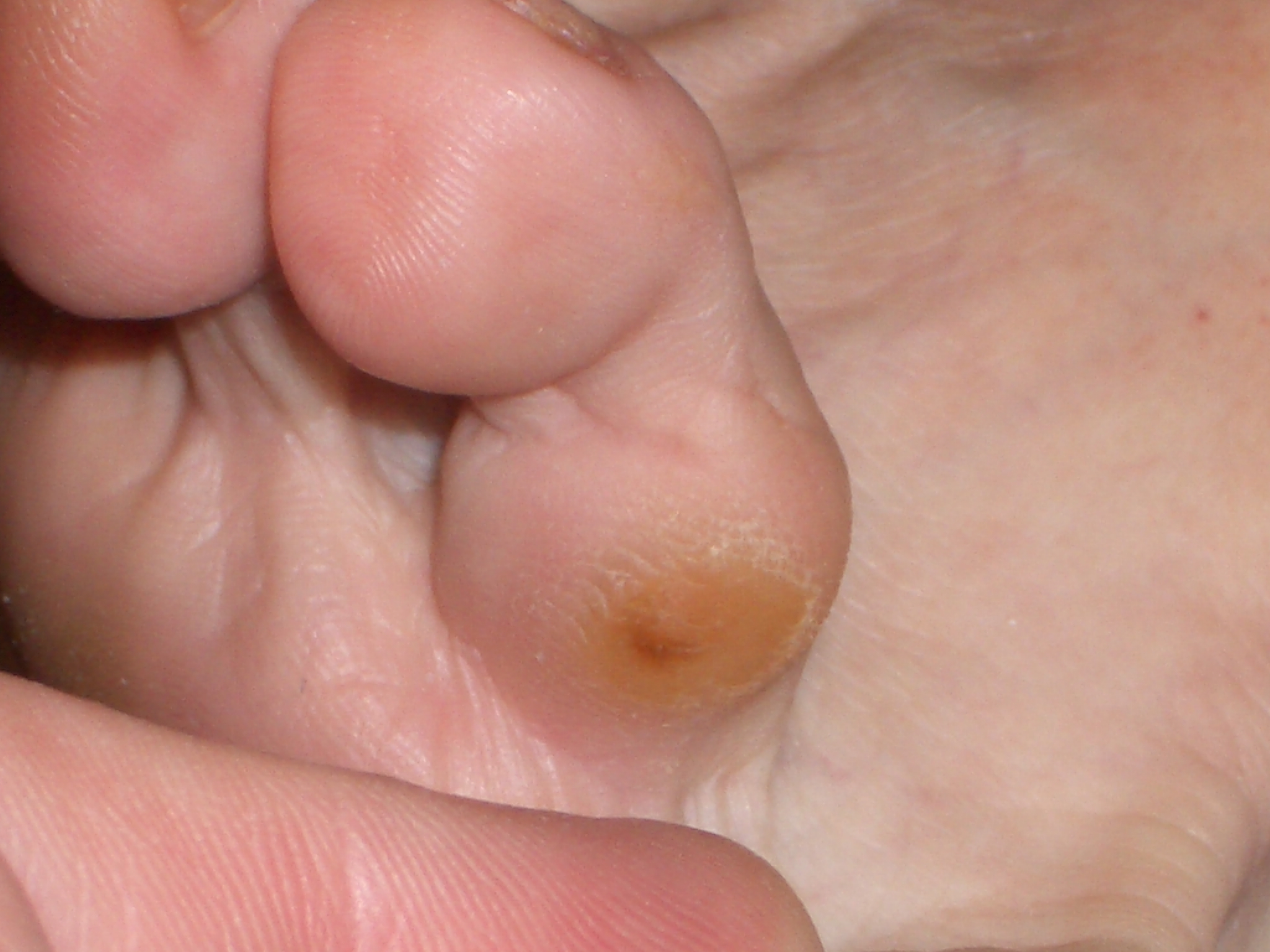

Supplement: Supplementary file 2 — Additional file 2: Figure S2. Heloma on head proximal phalanx on fourth toe. [file 13047_2021_460_MOESM2_ESM.jpg]

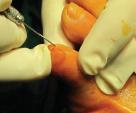

Supplement: Supplementary file 3 — Additional file 3: Figure S3. Condylectomy incision. [file 13047_2021_460_MOESM3_ESM.jpg]

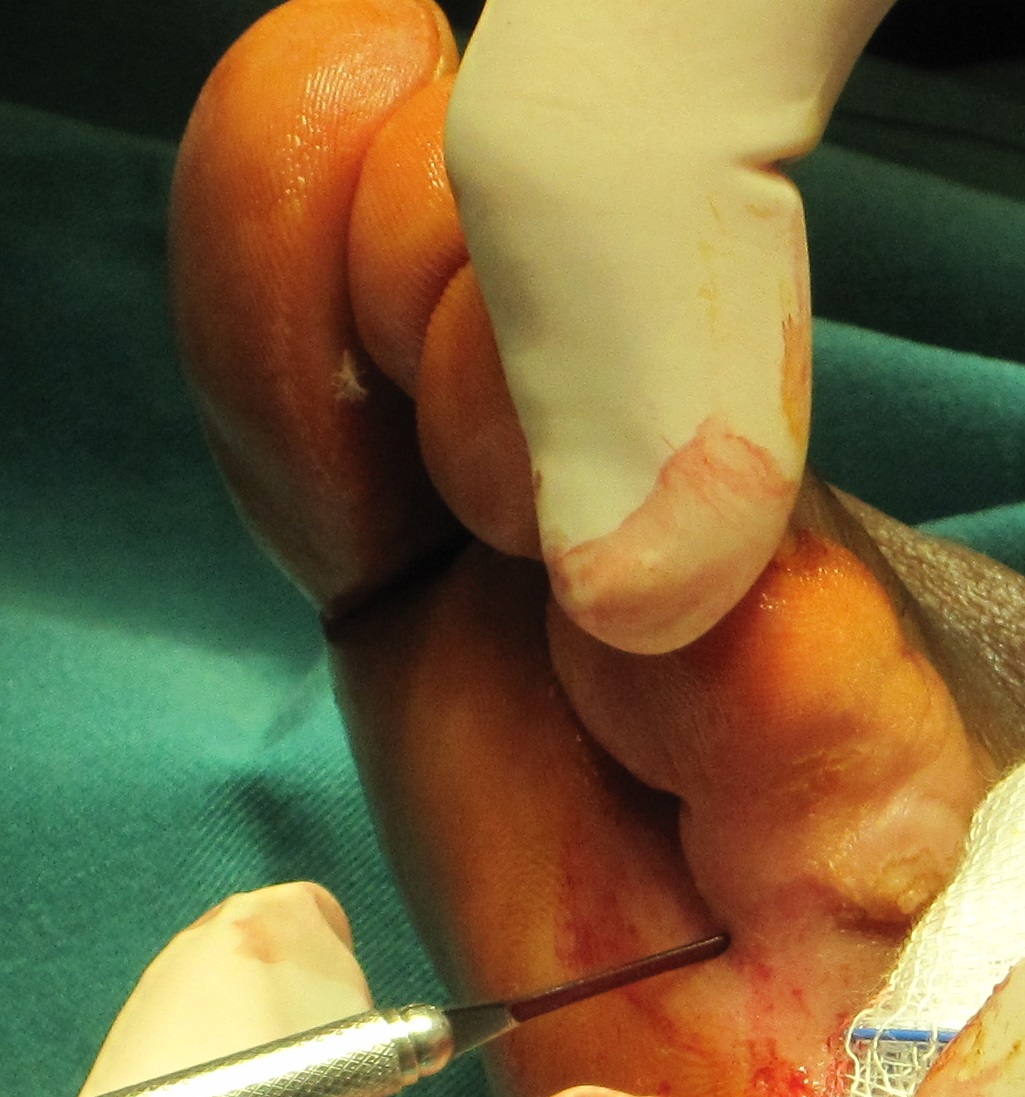

Supplement: Supplementary file 4 — Additional file 4: Figure S4. Condilectomy incision with Beaver 64 scalpel blade. [file 13047_2021_460_MOESM4_ESM.jpg]

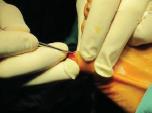

Supplement: Supplementary file 5 — Additional file 5: Figure S5. Blunt elevator to separate adhesions. [file 13047_2021_460_MOESM5_ESM.jpg]

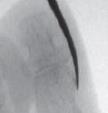

Supplement: Supplementary file 6 — Additional file 6: Figure S6. Fluoroscope image of separation of adhesions. [file 13047_2021_460_MOESM6_ESM.jpg]

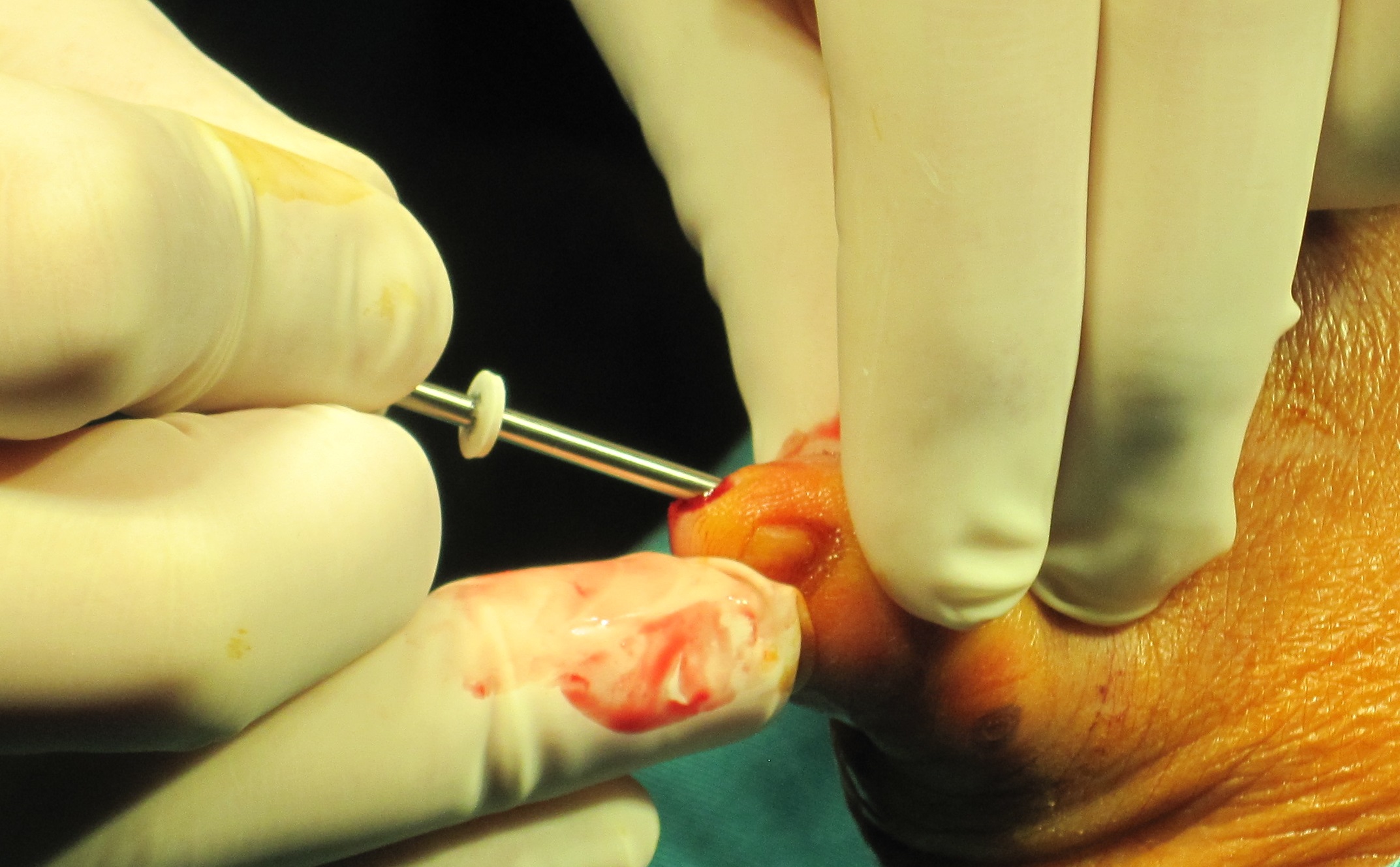

Supplement: Supplementary file 7 — Additional file 7: Figure S7. Osteotripsy with Shannon-Isham burr. [file 13047_2021_460_MOESM7_ESM.jpg]

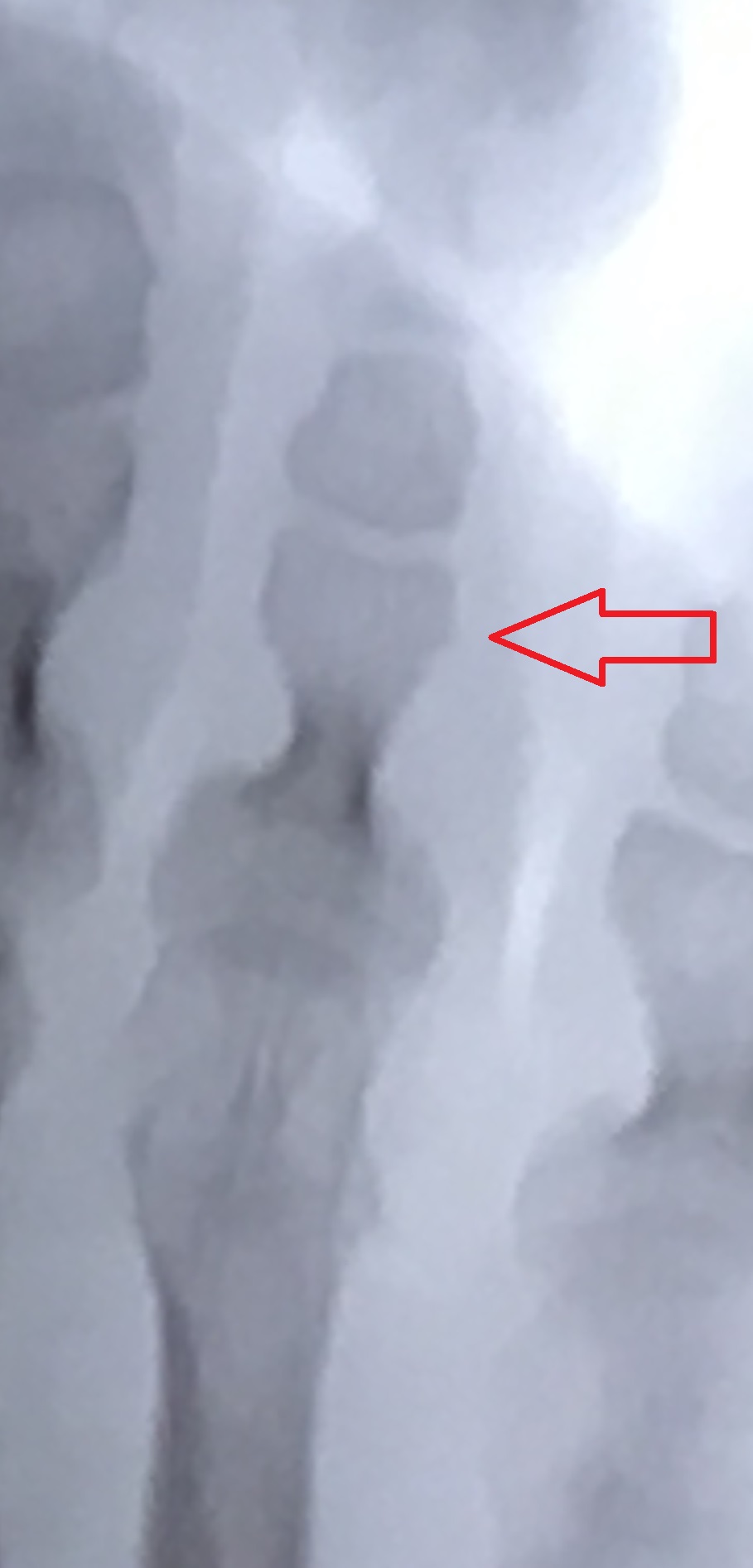

Supplement: Supplementary file 8 — Additional file 8: Figure S8. Fluoroscope image before condylectomy. [file 13047_2021_460_MOESM8_ESM.jpg]

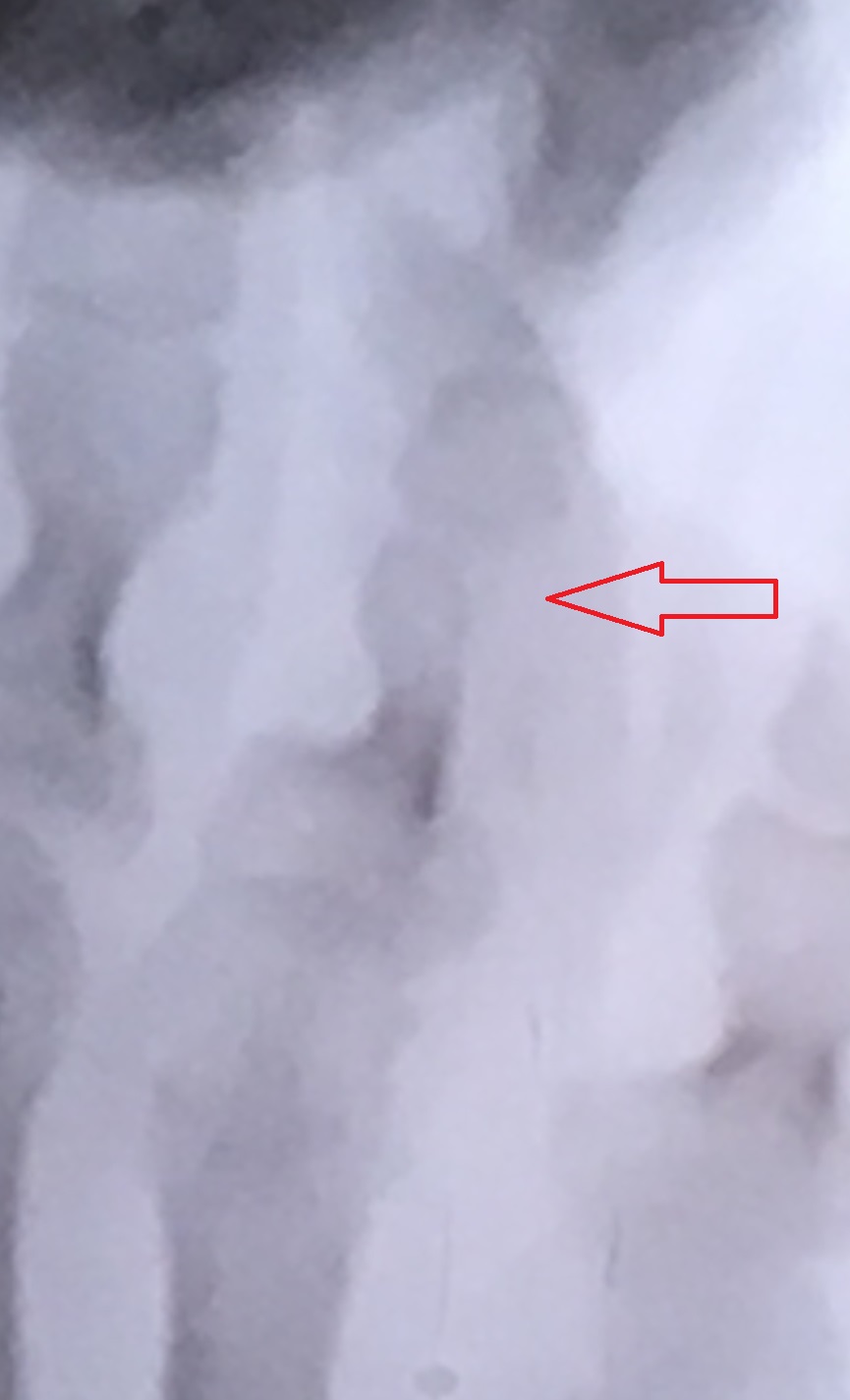

Supplement: Supplementary file 9 — Additional file 9: Figure S9. Fluoroscope image after condylectomy. [file 13047_2021_460_MOESM9_ESM.jpg]
