# Supplementary material for: Efficacy and safety of condylectomy with minimally invasive surgery in the treatment of interdigital corns of the lesser toes compared to conservative treatment
Source: J Foot Ankle Res. 2021 Mar 20;14:20. doi: 10.1186/s13047-021-00460-0 (PMC7981973; doi:10.1186/s13047-021-00460-0)
Supplement: Supplementary file 10 — Additional file 10: Table S1. Categories used to evaluate the degree of patient satisfaction with the treatment, based on a structured interview. [file 13047_2021_460_MOESM10_ESM.docx]

Table S1. Categories used to evaluate the degree of patient satisfaction with the treatment, based on a structured interview

| Excellent | The patient had no problem with the treated toe(s) |
| --- | --- |
| Very good | The patient had no or only mild pain in the toe or toes treated, and walked without difficulties |
| Good | The patient had mild pain or mild problems with the treated toe(s), walked with little or no difficulty, and would perform the same treatment again |
| Fair | The patient had moderate pain, some difficulty walking, and had doubts about the treatment performed |
| Poor | The patient was in pain and regretted having undergone the treatment |
